# Association of TM6SF2 rs58542926 T/C gene polymorphism with hepatocellular carcinoma: a meta-analysis

**DOI:** 10.1186/s12885-019-6173-4

**Published:** 2019-11-21

**Authors:** Shan Tang, Jing Zhang, Ting-Ting Mei, Hai-Qing Guo, Xin-Huan Wei, Wen-Yan Zhang, Ya-Li Liu, Shan Liang, Zuo-Peng Fan, Li-Xia Ma, Wei Lin, Yi-Rong Liu, Li-Xia Qiu, Hai-Bin Yu

**Affiliations:** Department of Hepatitis C and drug-induced liver injury, Beijing YouAn Hospital,Capital Medical University, Beijing, 100069 China

**Keywords:** TM6SF2 gene polymorphism, Liver cancer, Meta-analysis

## Abstract

**Background:**

Hepatocellular carcinoma (HCC) is the sixth-most common malignancy worldwide. Multiple previous studies have assessed the relationship between TM6SF2 gene polymorphism and the risk of developing HCC, with discrepant conclusions reached. To assess the association of TM6SF2 rs58542926 T/C gene polymorphism with liver cancer, we performed the current meta-analysis.

**Methods:**

This study queried the MEDLINE, PubMed, EMBASE, and CENTRAL databases from inception to April 2019. Case-control studies assessing the relationship between TM6SF2 rs5854292 locus polymorphism and liver cancer were selected according to inclusion and exclusion criteria. The Stata 12.0 software was employed for data analysis.

**Results:**

A total of 5 articles, encompassing 6873 patients, met inclusion criteria and were included in the meta-analysis. Statistical analysis showed that the TM6SF2 gene polymorphism was significantly associated with liver cancer in the allele contrast, dominant, recessive and over dominant models (T vs C, OR = 1.621, 95%CI 1.379–1.905; CT + TT vs CC. OR = 1.541, 95%CI 1.351–1.758; TT vs CT + CC, OR = 2.897, 95%CI 1.690–4.966; CC + TT vs TC, OR = 0.693, 95%CI 0.576–0.834). The Egger’s test revealed no significant publication bias.

**Conclusion:**

The present findings suggest a significant association of TM6SF2 gene polymorphism with HCC risk in the entire population studied.

## Background

Hepatocellular carcinoma ranks as the sixth-most common malignancy worldwide [1]. Recent cancer incidence data confirmed that the global age normalization rate (ASR) of primary liver cancer is 10.1/100,000, with a male/female ratio of 3:1 [2]. Hepatocellular carcinoma (HCC) diagnosis usually occurs in the late stages, resulting in elevated death rate; this makes HCC the third deadliest malignancy [3]. A single nucleotide polymorphism (SNP) is a result of transition or transversion mutation of a single base, and is significantly associated with various genetic diseases [4]. Moreover, current studies have initially demonstrated that different SNPs have different roles in liver damage, and some of them increase the risk of chronic liver disease and HCC through genetic variation alone or in combination with clinical variables [5]. The role of a common non-synonymous polymorphism in transmembrane 6 superfamily member 2 (rs58542926 c.449 C > T, p.Glu167Lys, E167K) in lipid metabolism and chronic liver disease has attracted attention, with multiple studies focused on the role of TM6SF2 rs58542926 variant in chronic liver disease and HCC [6, 7]. Genotyping will allow for more precise HCC risk-stratification of patients with chronic liver diseases, and genotype-guided screening algorithms would optimize patient care [8]. Assessing genetic risk factors associated with development of HCC may allow for earlier diagnosis of malignancy and could potentially lead to decreased disease-specific mortality rates. Current studies have shown that transmembrane 6 superfamily member 2 (TM6SF2) rs5854292 gene polymorphism is associated with nonalcoholic fatty liver disease [9, 10]. Meanwhile, multiple investigators in China and abroad have carried out a large number of studies to assess the relationship between TM6SF2 rs5854292 gene polymorphism and liver cancer. Some studies concluded that the TM6SF2 rs5854292 variant was associated with the risk of developing [11]. Other studies, however, demonstrated that the presence of the TM6SF2 variant did not appear to be associated with further increased risk of developing HCC [12]. To further clarify the relationship between TM6SF2 rs58542926 gene polymorphism and liver cancer,we conducted this meta-analysis of published research.

## Methods

The current meta-analysis complied with Preferred Reporting Items for Systematic Reviews and Meta-Analyses (PRISMA) guidelines [13]. The search strategy, eligibility criteria and outcomes were described a priori (PROSPERO CRD42019126384).

### Data sources and search strategies

A comprehensive search for literature addressing the genetic associations of TM6SF2 variants in patients with HCC was conducted in the Medline, EMBASE, PubMed and CENTRAL databases without language restriction, from inception to April 2019. The specific search strategy was “ Liver Neoplasms or Hepatic Neoplasms or Hepatocellular Cancer or Liver Cancer” and “TM6SF2 protein or Transmembrane 6 superfamily member 2”. Table 1 summarizes the search strategy for PubMed, and it was also employed for all databases. The last literature search in the above databases was completed on April 11, 2019.

**Table 1:** PubMed search strategy

Number	Search items
#1	“Liver Neoplasms” [Mesh]
#2	(Neoplasms, Liver OR Liver Neoplasm OR Hepatic Neoplasms OR Hepatic Neoplasm OR Cancer of Liver OR Hepatocellular Cancer OR Hepatocellular Cancers OR Hepatic Cancer OR Hepatic Cancers OR Liver Cancer OR Cancers, Liver OR Cancer of the Liver)
#3	(Liver*Neoplasm*OR Hepat*Neoplasm*OR Liver*Cancer*OR Hepat*Cancer*) [Title/Abstract]
#4	#1 OR #2 OR #3
#5	“TM6SF2 protein, human” [Supplementary Concept]
#6	(Transmembrane 6 superfamily member 2 OR TM6SF2 OR E167K OR rs58542926) [Title/Abstract]
#7	#5 OR #6
#8	#4 AND #7

### Study selection

Relevant articles were initially selected based on title and abstract. Then, two authors reviewed the full texts to select qualified articles based on set eligibility criteria. Any disputes during the selection process were discussed with and resolved by a third investigator.

### Inclusion and exclusion criteria of the literature

**Inclusion criteria:** **(**1) the study cohorts included TM6SF2 rs58542926 T/C gene polymorphism in patients with liver cancer and non-hepatoma individuals; (2) histological features were assessed by liver biopsy, and diagnostic criteria were clearly stated; (3) case-control studies were enrolled, and the control group included non-hepatoma cases; (4) if two (or more) studies included the same cohort, the most recent was included to avoid repeated statistics; (5) the risk ratios of hazardous variants on the susceptibility of hepatocellular carcinoma were reported or could be calculated; (6) the full text could be retrieved by different ways. **Exclusion criteria:** (1) the source of enrolled cases in the article is unclear;(2) no clear diagnostic criteria for HCC described; (3) data collection and analysis methods unscientific or inappropriate; (4) lack of detailed genotyping data; (5) no-case-control study; (6) *in* animal studies.

### Data extraction

Two experienced authors independently extracted the necessary data and information from eligible publications according to a predetermined data extraction form. The information extracted from all the selected studies included: first author’s surname, publication year, country in which the study was conducted, total numbers of patients in the case and control groups, sex ratio, age, and body mass index (BMI), as well as the numbers of cases and controls with the C/C, C/T, and T/T genotypes. Whether genotype distribution was consistent with the Hardy-Weinberg equilibrium (HWE) was also recorded.

### Risk of bias

The Egger’s test was used for assessing publication bias, with *P* < 0.05 considered to present statistical significance.

### Statistical analysis

The association of the T/C polymorphism in the TM6SF2 gene with HCC susceptibility was evaluated by calculating pooled odds ratios (ORs) alongside 95% confidence intervals (CIs) in the allelic, dominant, recessive, and super-dominant models. The HWE for each study was measured by the *χ*^2^ test, and *P* > 0.05 was regarded as consistent with the HWE. The random or fixed effects model was used to pool ORs based on heterogeneity assumption, Heterogeneity across studies was determined by the Q- and *I*^2^ tests. The fixed-effects model was used in the case of nonsignificant heterogeneity (*P* > 0.05, *I*^2^ < 50%); otherwise, the random-effects model was utilized. In order to explore the effect of a single study on overall results, sensitivity analysis was performed by removing one study sequentially to evaluate its effect on the overall results under all genetic models. The STATA 12.0 software was employed for statistical analyses.

## Results

### Search results

**Fig. 1:**
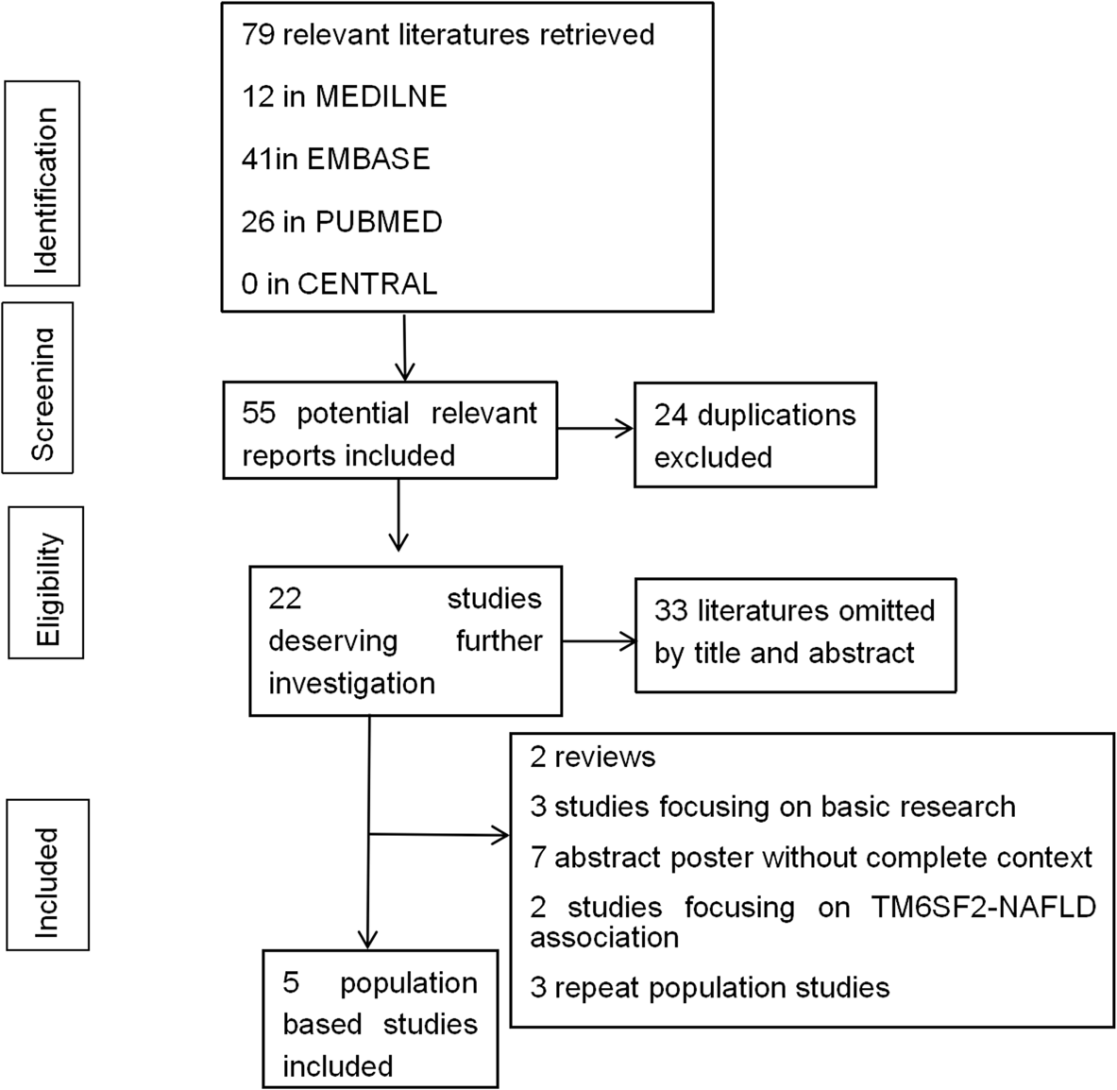
Flow diagram for study selection

There were a total of 79 relevant studies compliant with the strategy, of which 24 were excluded as duplicates. After further title and abstract review, 33 reports were excluded as irrelevant to this meta-analysis. The second-round of review was based on careful full-text review of the 22 retained papers. Then, 17 reports were exclude, leaving 5 that were included in the final analysis. Figure 1 summarizes the above selection process.

### Characteristics of the included studies

Table 1 depicts the basic features of all five included studies. A total of 2594 patients with liver cancer (case group) and 4279 patients without hepatocarcinoma (control group) were included. Genotyping data for all studies are summarized in Table 1. The vast majority of reports used TaqMan assays for genotyping. One of the studies did not provide specific frequency distributions of CC, CT, and TT, and only provided the frequency distribution in the dominant model (CT + TT vs CC). The assessed individuals were mostly Europeans and Asians. One of the reports had the control group’s genotype distribution deviating from the HWE.

### Meta-analysis results

Five studies included in the current meta-analysis described the association of TM6SF2 rs58542926 T/C gene polymorphism with susceptibility to liver cancer. The allelic (T vs C), dominant (CT + TT vs CC), recessive (TT vs CT + CC), and super-dominant (CC + TT vs TC) models were assessed. Since one of the studies provided no specific frequency distributions for CC, CT, and TT, and only provided frequency distribution in the dominant model (CC vs CT + TT), the five reports were included for assessment in the dominant model. In the allelic, recessive and super-dominant models, 4 studies were included, with a total of 2462 HCC patients and 3464 controls. The fixed effects model was employed for pooled ORs since nonsignificant heterogeneity was detected. The results showed that the TM6SF2 gene polymorphism was significantly associated with susceptibility to liver cancer (Table 2).

**Table 2:** Characteristics of the studies included in the meta-analysis

Study	Cohort characteristics	Sample size	genotype			allele	
			TT	TC	CC	T	C
Edmondo Falleti, 2015^[14]^	HCC patients	150	1	26	123	28	272
	cirrhosis patients	361	1	40	320	42	680
Maneerat Raksayot, 2018^[15]^	HCC patients	541	10	134	397	154	928
	healthy controls	105	1	15	89	17	193
Felix Stickel, 2018^[16]^	HCC patients	751	29	164	558	222	1280
	alcohol-related cirrhosis	1165	15	193	957	223	2107
Jie Yang, 2018^[17]^	HCC patients	1020	210		810	-	-
	chronic liver disease	2015	300		1715	-	-
Benedetta Donati, 2017^[18]^	HCC patients	132	4	19	109	27	237
	NAFLD	633	7	88	538	102	1164

### TM6SF2 rs58542926 T/C in the dominant model (CT + TT vs CC)

**Fig. 2:**
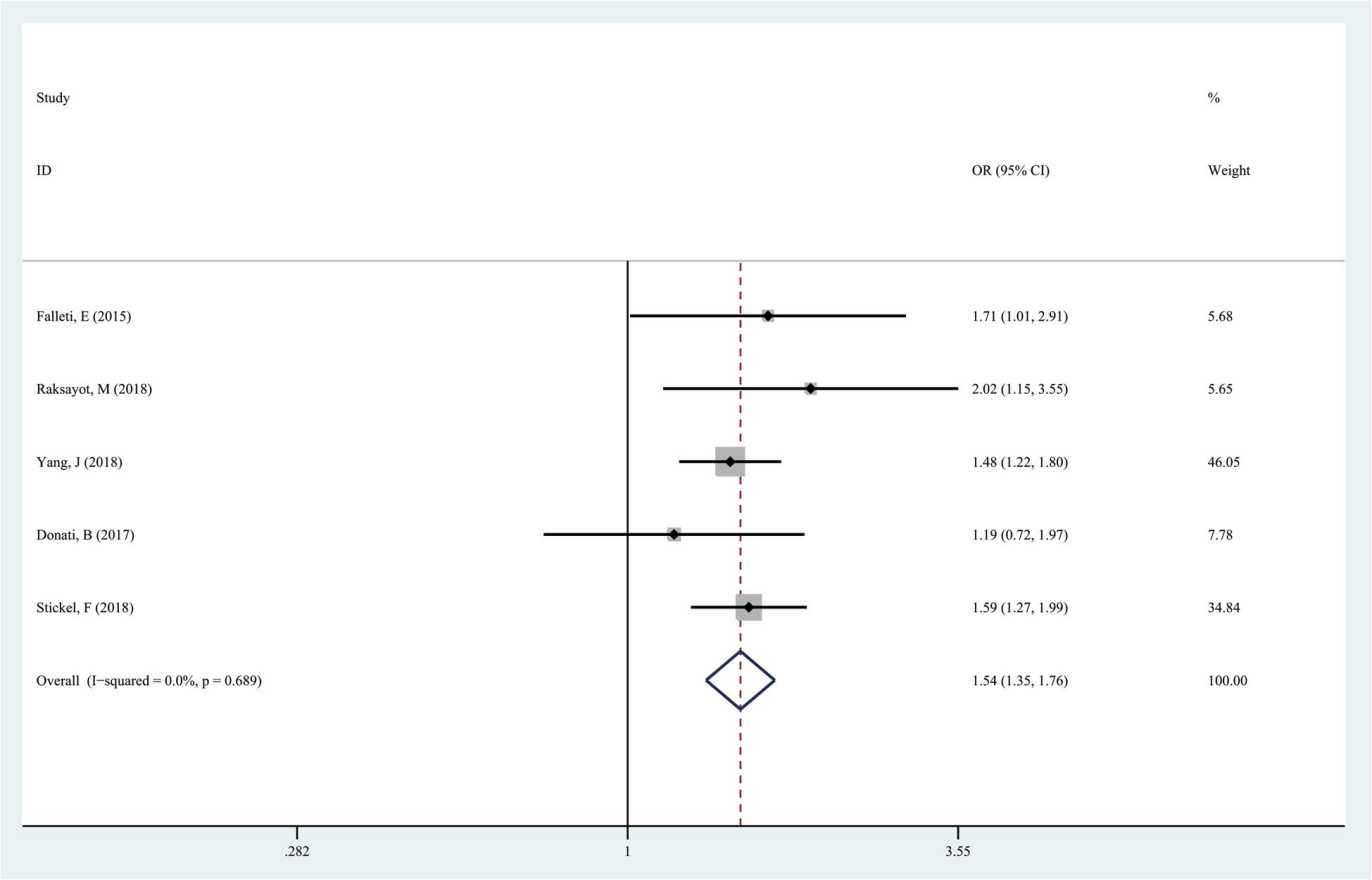
Forest plot of studies evaluating the OR with 95%CI of TM6SF2 rs58542926 T/C in the dominant model (CT + TT vs CC) in liver cancer patients. CI, Confidence interval; OR, odds ratio

The CT + TT genotype as the exposure factor and the CC genotype as the non-exposure factor were analyzed. A total of 597 and 1997 cases had the TT + CT and CC genotypes in the case group, respectively. Meanwhile, 660 and 3619 cases had the TT + CT and CC genotypes in the control group, respectively. The results showed that the pooled risk of liver cancer was higher in the TT + CT genotype compared with the CC genotype (CC vs CT + TT, OR = 1.541; 95%CI 1.351–1.758; *P* = 0.000; Fig. 2).

### TM6SF2 rs58542926 T/C in the allelic model (T vs C)

**Fig. 3:**
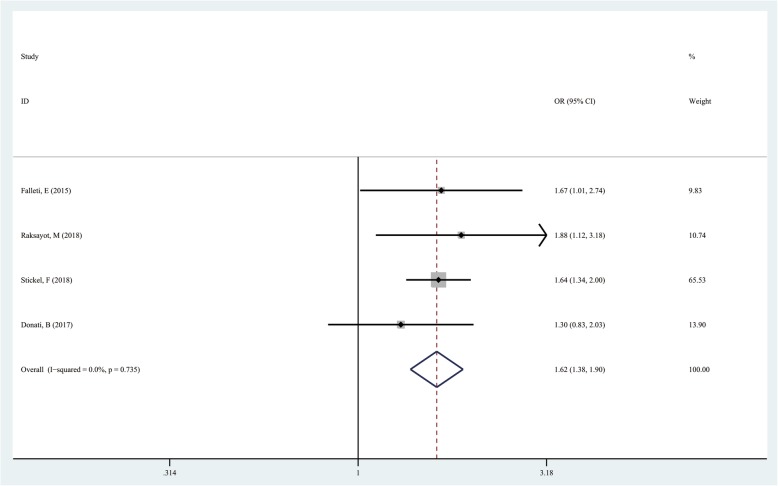
Forest plot of studies evaluating the OR with 95%CI of TM6SF2 rs58542926 T/C in the allelic model (T vs C) in liver cancer patients. CI, Confidence interval; OR, odds ratio

The T allele was used as the exposure factor and the C allele as the non-exposure factor. There were 431 cases with the T allele and 2771 with the C allele in the case group, and 384 T allele and 4144 C allele cases in the control group. We found that TM6SF2 rs58542926 T/C gene polymorphism had a significant association with hepatocellular carcinoma (T vs C, OR = 1.621; 95%CI 1.379–1.905; *P* = 0.000; Fig. 3).

### TM6SF2 rs58542926 T/C in the recessive model (CT + CC vs TT)

**Fig. 4:**
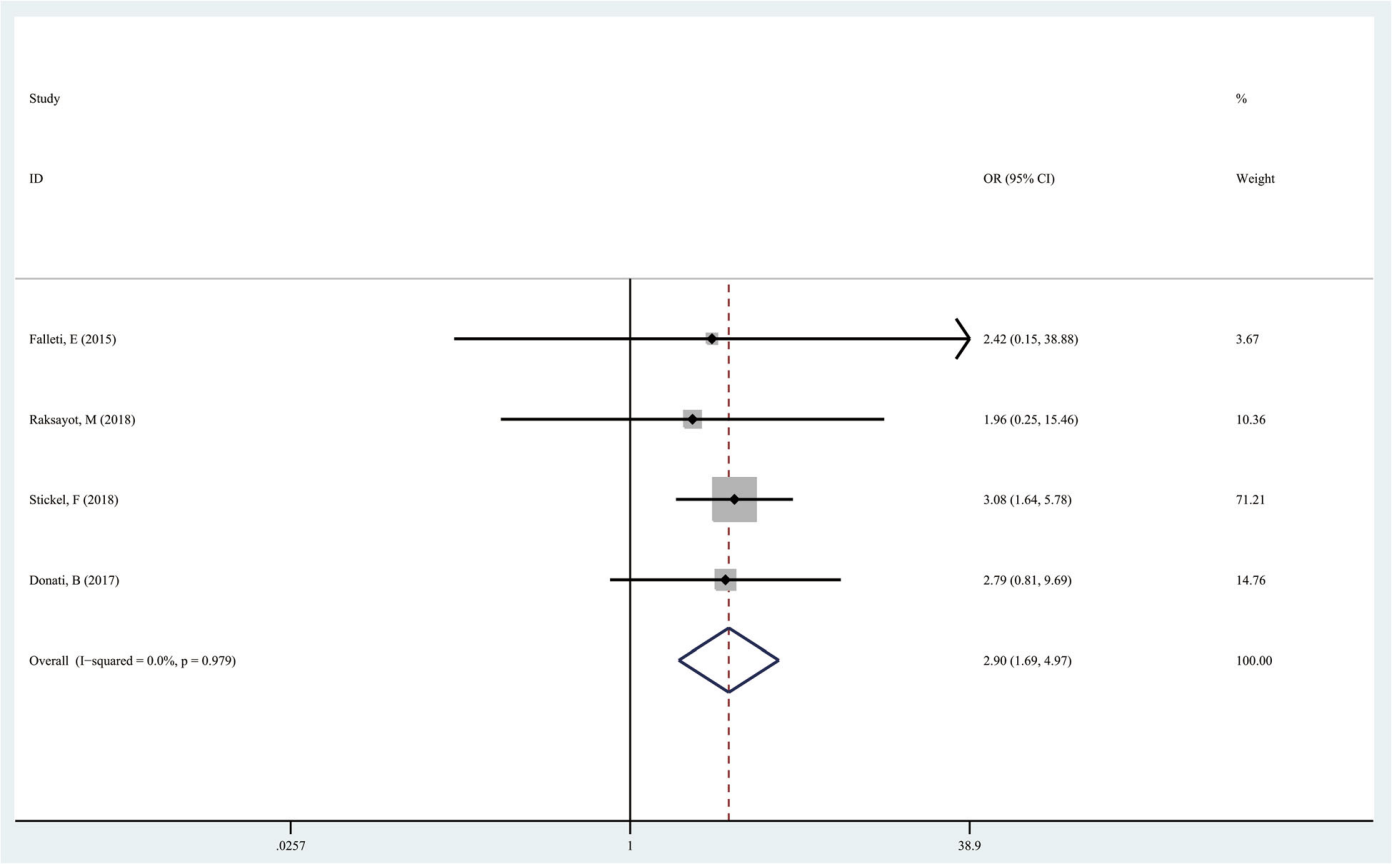
Forest plot of studies evaluating the OR with 95%CI of TM6SF2 rs58542926 T/C in the recessive model (CC + CT vs TT) in liver cancer patients. CI, Confidence interval; OR, odds ratio

The TT genotype was used as the exposure factor and the CC + CT genotype as the non-exposure factor. A total of 44 patients had the TT genotype and 1530 displayed the CC + CT genotype among cases. Meanwhile, 24 and 2240 cases had the TT and CC + CT genotypes among controls, respectively. The results showed that the risk of liver cancer in the TT genotype group was higher than that of the CC + CT genotype group (TT vs CT + CC, OR = 2.897; 95%CI 1.690–4.966; *P* = 0.000; Fig. 4).

### TM6SF2 rs58542926 T/C in the super-dominant model (CC + TT vs TC)

**Fig. 5:**
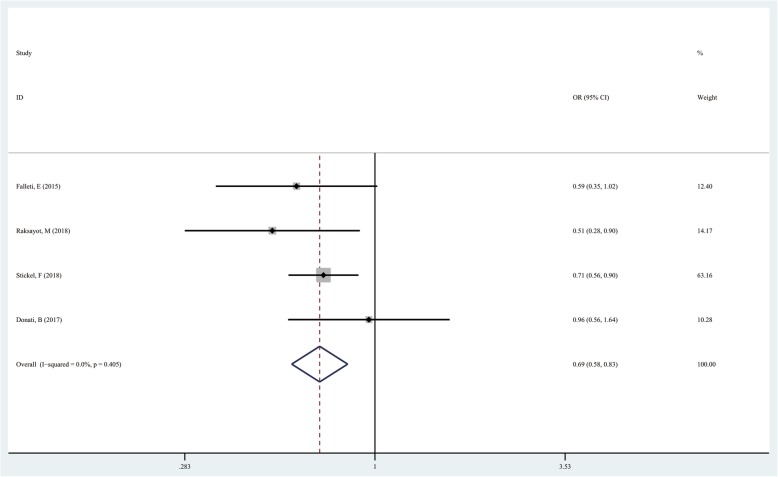
Forest plot of studies evaluating the OR with 95%CI of TM6SF2 rs58542926 T/C in the super-dominant model (CC + TT vs TC) in liver cancer patients. CI, Confidence interval; OR, odds ratio

The TT + CC genotype was used as the exposure factor and the CT genotype as the non-exposure factor. There were 1231 cases with the TT + CC genotype and 323 with the CT genotype in the case group. Meanwhile, 1928 and 336 cases had the TT + CC and CT genotypes in the control group, respectively. The results showed that the risk of liver cancer in the TT + CC genotype groups was lower than that of individuals with the CT genotype (CC + TT vs TC, OR = 0.693; 95%CI 0.576–0.834; *P* = 0.000; Fig. 5) (Table 3).

**Table 3:** Meta-analysis of the association of TM6SF2 rs58542926 T/C gene polymorphism with hepatocellular carcinoma susceptibility

Genetic model	Relevance test			Heterogeneity test			Publication bias	
	OR(95%CI)	Z	*P*_value_	*I*^2^	Q	*P*_hel_	*P*_egger_	t
dominant gene model	1.541 (1.351–1.758)	6.44	0.000	0	2.26	0.689	0.728	0.38
Allelic model	1.621 (1.379–1.905)	5.86	0.000	0	1.28	0.735	0.904	−0.14
recessive model	2.897 (1.690–4.966)	3.897	0.000	0	0.19	0.979	0.120	−2.63
super-dominant model	0.693 (0.576–0.834)	3.87	0.000	0	2.91	0.405	0.776	−0.32

### Sub-analysis

To further clarify whether the different causes of liver cancer affect the results of the meta-analysis, we divided the causes of HCC into viruses, NAFLD, and alcoholic liver disease. There are 3 articles on the relationship between TM6SF2 gene polymorphism and HCC caused by alcoholic liver disease. The results of META analysis on dominant gene model showed that the pooled risk of liver cancer was higher in the TT + CT genotype compared with the CC genotype (CC vs CT + TT, OR = 1.675; 95% CI 1.413–1.985; *P* = 0.000), sensitivity analysis suggested that the result was relatively stable. There are two articles on the relationship between TM6SF2 gene polymorphism and HCC caused by viral hepatitis. The results of META analysis on the dominant gene model showed that the pooled risk of liver cancer was higher in the TT + CT genotype compared with the CC genotype (CC vs CT + TT, OR = 1.491; 95% CI 1.048–2.122; *P* = 0.026), sensitivity analysis suggested that the result was relatively robust. There is one article to study the relationship between TM6SF2 gene polymorphism and NAFLD-induced HCC, so there is no META analysis result. Due to the limitation of the number of articles included, the results of stratified analysis of HCC by cause are not satisfactory, but according to the current analysis results, the cause of HCC seems to have no effect on the meta analysis. There are also some documents that confirm this claim. Stickel F et al. showed that the development of HCC was independently associated with TM6SF2 rs58542926, Carriage of TM6SF2 rs58542926 is an independent risk factor for the development of HCC in people with alcohol-related cirrhosis [8]. More recently, the TM6SF2 polymorphism was characterized among the independent predictors of NAFLD-HCC even after adjustment for age, sex, T2DM and advanced fibrosis [5, 11].

### Sensitivity analysis

Sensitivity analysis was performed by omitting one study sequentially to examine its effect on the overall results under all genetic models. In the four genetic models of TM6SF2 rs58542926 T/C, OR values obtained after eliminating any one of the studies were close to pre-exclusion ORs, indicating the robustness of the current analysis (Additional file 1: Figures S1, S2, S3, and S4).

### Publication bias

Egger’s funnel plots showed that the meta-analysis had no publication bias in the four genetic models, including the allelic (T vs C, *P* = 0.728), dominant (CT + TT vs CC, *P* = 0.904), recessive (TT vs CT + CC, *P* = 0.120) and super-dominant (CC + TT vs TC, *P* = 0.776) models (Additional file 1: Figures S5, S6, S7, and S8).

## Discussion

The TM6SF2 gene E167K variant (rs58542926) features a guanine to adenine substitution (nucleotide position 499), resulting in glutamate to lysine change at amino acid position 167 (E167K) [19]. Subcellular localization analysis showed that TM6SF2 is mainly expressed in the intermediate compartment of the endoplasmic reticulum (ER) and ER-Golgi intermediate in HepG2 cells [19]. TM6SF2 represents an ER membrane protein, and E167K mutation results in cell division and enhances TM6SF2 biodegradation [20]. Toll-like receptor secretion from and liver lipid droplet amounts in HepG2 cells are affected by TM6SF2 downregulation [20]. The TM6SF2 E167K variant might influence the cell cycle in HCC HEPA1–6 cells via cyclin D1 and P53 upregulation and P27 downregulation [21]. Dysregulated cell cycle alters energy metabolism and within hepatocytes, may be associated with increased risk of the development of HCC [21].

Recent studies have shown thatTM6SF2 may not solely be a marker associated with increased risk of HCC, but may be involved in the development of HCC at the cellular level. Shuixian Du et al. investigated the effect of TM6SF2 E167K on the expression levels of TNF-α, IL-2, IL-6 and IL-8 in the HCC cell HEPA 1–6,and demonstrated that overexpression of the TM6SF2 E167K protein significantly up-regulates the expression of IL-2 and IL-6 [22]. Their findings suggest that the TM6SF2 E167K variant could promote the inflammatory response and aggravated cell injury observed in HCC.

Although the relationship between TM6SF2 gene polymorphism and the risk of liver cancer has attracted attention from many researchers, results vary from study to study. In a single study, the stability and reliability of the research results are affected by the limited sample size. However, meta-analyses use suitable mathematical models to perform quantitative analysis of multiple identical or similar research results, increasing the test efficiency of research results.

Here, a search strategy was designed, and article quality was assessed based on the Oxford Critical Appraisal Skill Program guidelines (Oxford CASP, 2004) [23]. Articles with no-control group or unclear diagnostic criteria, as well as duplicated reports were excluded. Finally, 5 articles that met the set requirements were included for data extraction. Applying the fixed-effects model to pool research data from different locations obtained at distinct times to analyze the relationship between TM6SF2 rs58542926 locus polymorphism and liver cancer. We employed the allelic, dominant, recessive and super-dominant models of TM6SF2 rs58542926 for analysis. The results showed that TM6SF2 rs58542926 gene polymorphism was significantly associated with liver cancer susceptibility.

In sensitivity analysis, the meta-analysis findings were relatively stable. Publication bias is an important factor affecting the results of a meta-analysis. The Egger regression method was used to demonstrate that the meta-analysis had no overt publication bias, suggesting that the above results were reliable.

The limitations of this study could not be ignored. Firstly, due to the limited number of articles included, this meta-analysis failed to stage the tumors for group discussion. Secondly, this meta-analysis only involved single factor studies,the interactions of TM6SF2 gene polymorphisms and environmental factors, obesity, alcohol intake, intake of the fungal metabolite aflatoxin, and hepatitis B and C infections were not taken into consideration [24]. Meanwhile, the latter factors could influence susceptibility to hepatocellular carcinoma. Thirdly, the case groups all were made up of patients with a diagnosis of HCC, the pooled control group created from the 5 case-control studies included in the meta-analysis included both patients with chronic liver disease as well as healthy controls. This may have an impact on the credibility of the results of the meta-analysis. However, due to the limitation of the number of articles, this defect is difficult to overcome.

## Conclusion

In summary, TM6SF2 rs58542926 gene polymorphism is significantly associated with liver cancer susceptibility. It should be further investigated whether the TM6SF2 rs58542926 variant could be screened for early diagnosis of liver cancer.

## Supplementary information

**Additional file 1: Figure S1.** Sensitivity analysis of TM6SF2 rs58542926 T/C in the dominant model. **Figure S2** Sensitivity analysis of TM6SF2 rs58542926 T/C in the allelic model. **Figure S3.** Sensitivity analysis of TM6SF2 rs58542926 T/C in the recessive model. **Figure S4.** Sensitivity analysis of TM6SF2 rs58542926 T/C in the super-dominant model. **Figure S5.** Egger’s funnel plot of TM6SF2 rs58542926 T/C in the dominant model. **Figure S6.** Egger’s funnel plot of TM6SF2 rs58542926 T/C in the allelic model. **Figure S7.** Egger’s funnel plot of TM6SF2 rs58542926 T/C in the recessive model. **Figure S8.** Egger’s funnel plot of TM6SF2 rs58542926 T/C in the super-dominant model.

## Data Availability

All data generated or analyzed during this study are derived from previously published original research articles. Details are available from the corresponding author on reasonable request.

## Abbreviations

HCC: hepatocellular cancer
SNP: single nucleotide polymorphism
TM6SF2: transmembrane 6 superfamily member 2
