# Supplementary material for: Association of TM6SF2 rs58542926 T/C gene polymorphism with hepatocellular carcinoma: a meta-analysis
Source: BMC Cancer. 2019 Nov 21;19:1128. doi: 10.1186/s12885-019-6173-4 (PMC6868855; doi:10.1186/s12885-019-6173-4)
Supplement: Supplementary file 1 — Additional file 1: Figure S1. Sensitivity analysis of TM6SF2 rs58542926 T/C in the dominant model. Figure S2 Sensitivity analysis of TM6SF2 rs58542926 T/C in the allelic model. Figure S3. Sensitivity analysis of TM6SF2 rs58542926 T/C in the recessive model. Figure S4. Sensitivity analysis of TM6SF2 rs58542926 T/C in the super-dominant model. Figure S5. Egger’s funnel plot of TM6SF2 rs58542926 T/C in the dominant model. Figure S6. Egger’s funnel plot of TM6SF2 rs58542926 T/C in the allelic model. Figure S7. Egger’s funnel plot of TM6SF2 rs58542926 T/C in the recessive model. Figure S8. Egger’s funnel plot of TM6SF2 rs58542926 T/C in the super-dominant model. [file 12885_2019_6173_MOESM1_ESM.doc]

**
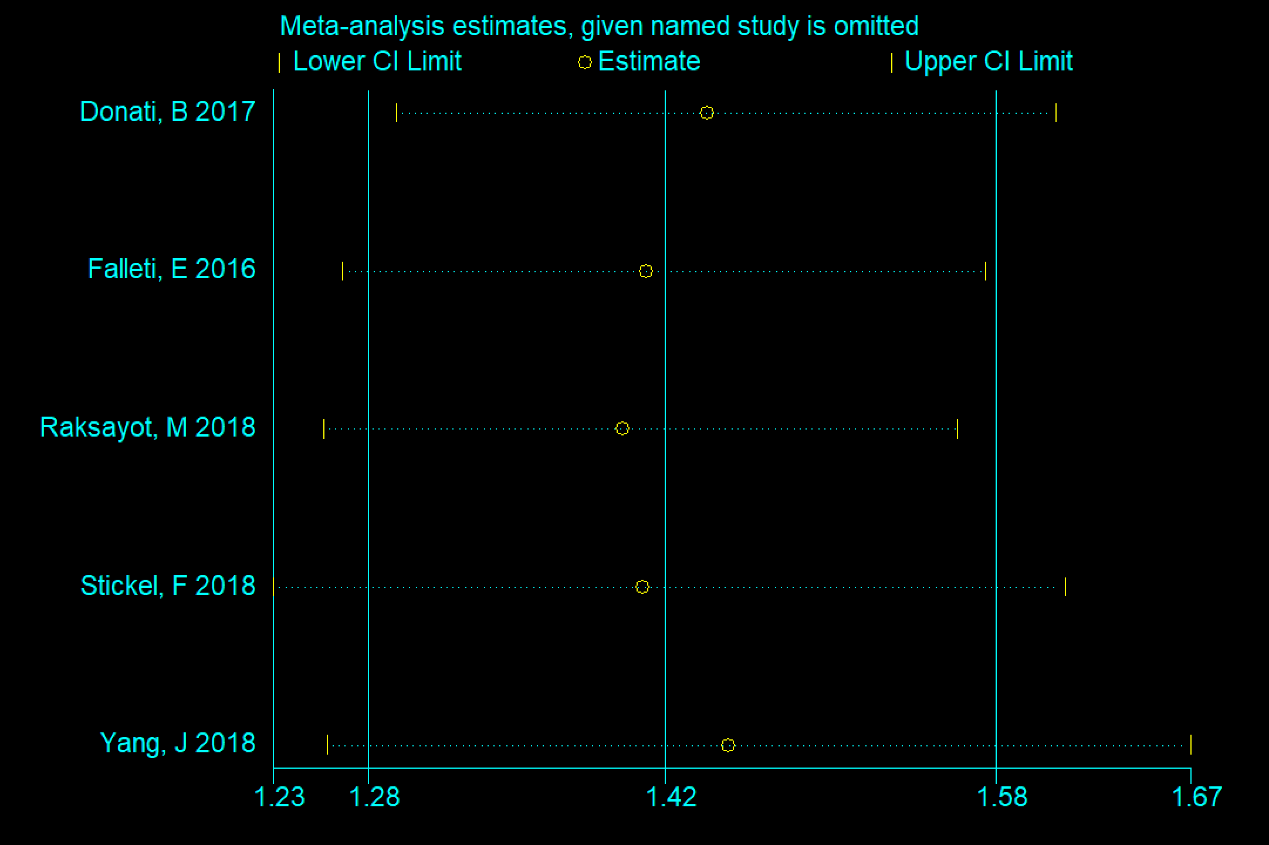
**

Supplementary Figure1 Sensitivity analysis of TM6SF2 rs58542926 T/C in the dominant model


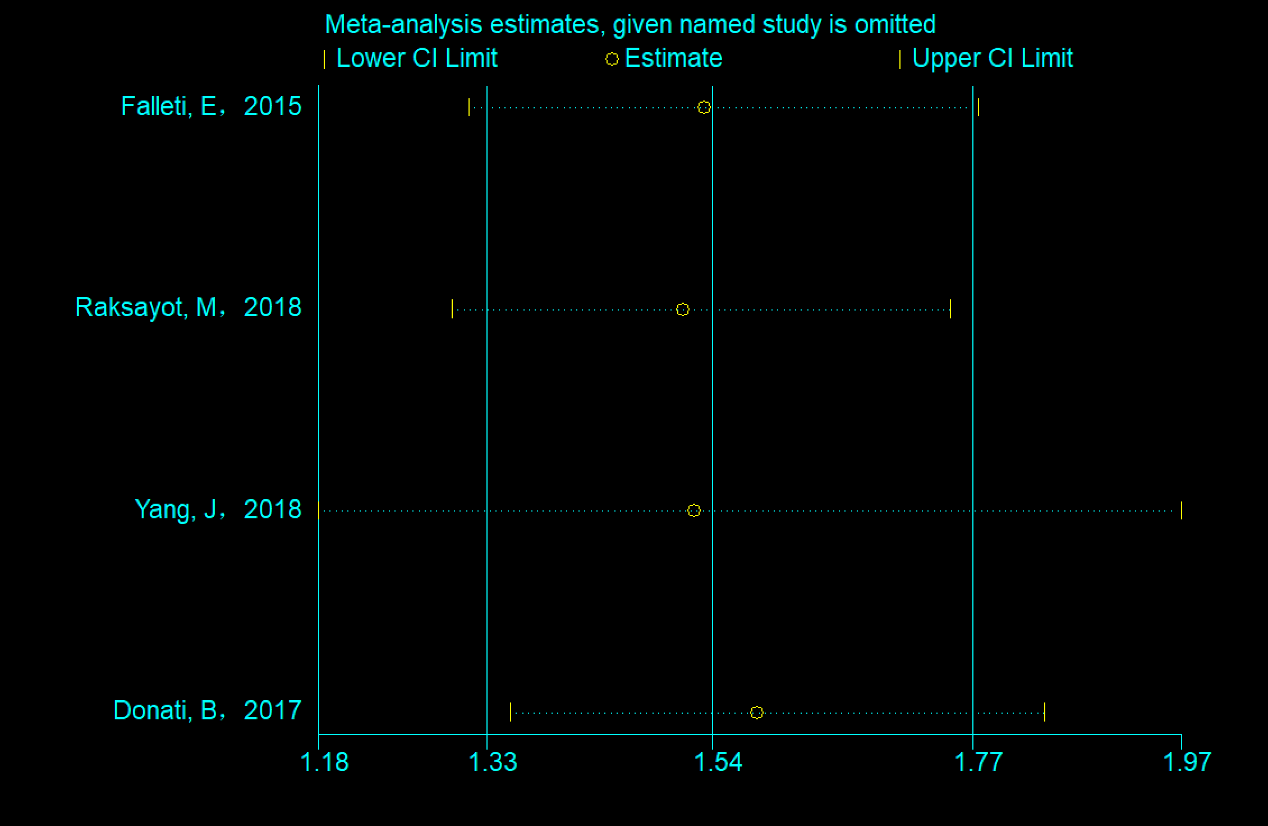


Supplementary Figure 2 Sensitivity analysis of TM6SF2 rs58542926 T/C in the allelic model


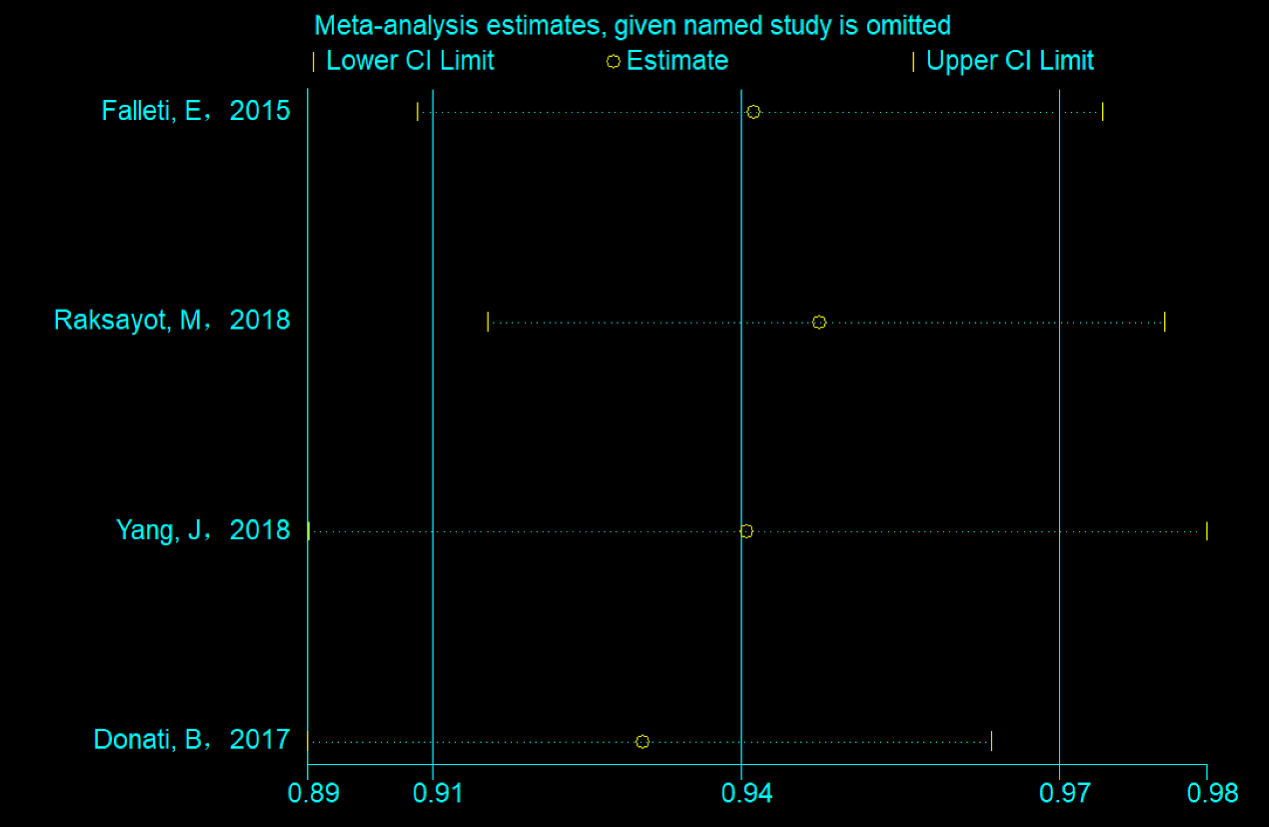


Supplementary Figure 3 Sensitivity analysis of TM6SF2 rs58542926 T/C in the recessive model


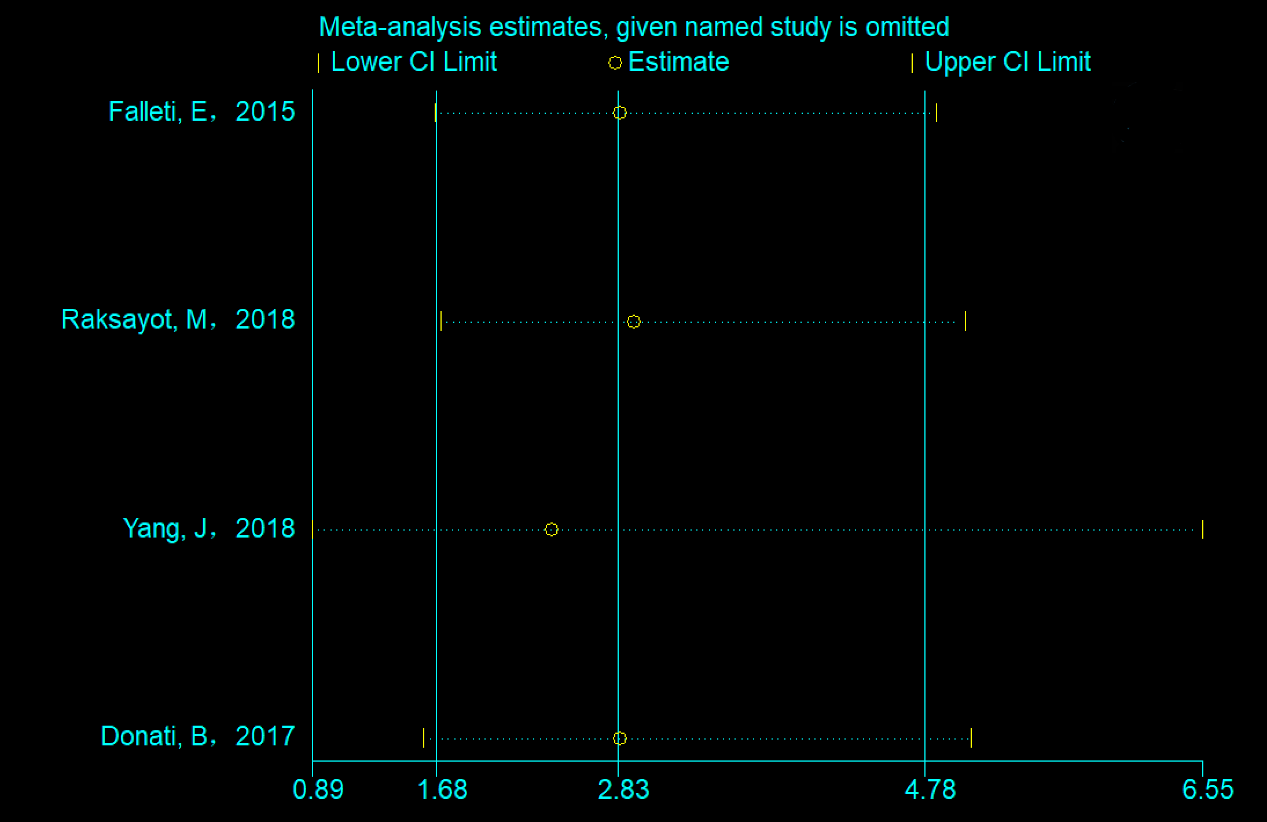


Supplementary Figure 4 Sensitivity analysis of TM6SF2 rs58542926 T/C in the super-dominant model

**Figure10**

**
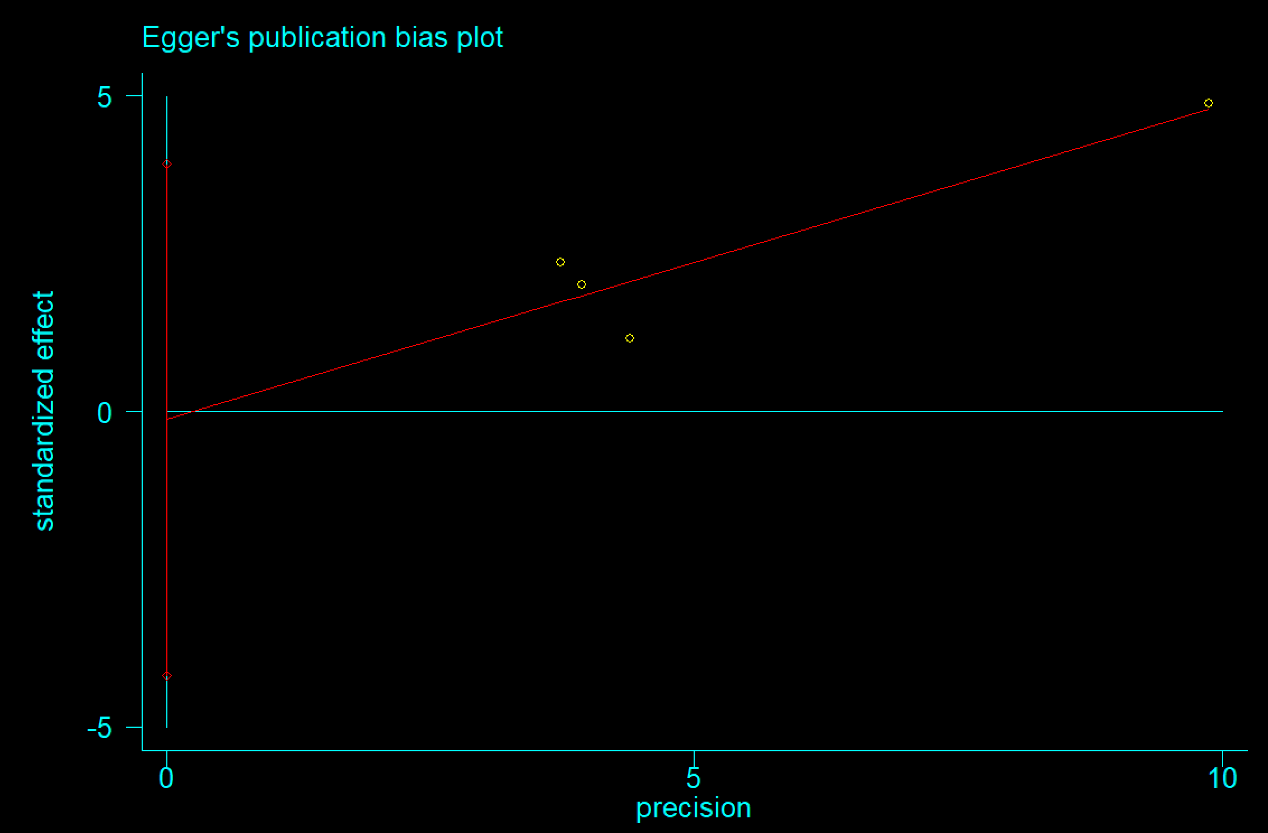
**

Supplementary Figure 5 Egger's funnel plot of TM6SF2 rs58542926 T/C in the dominant model


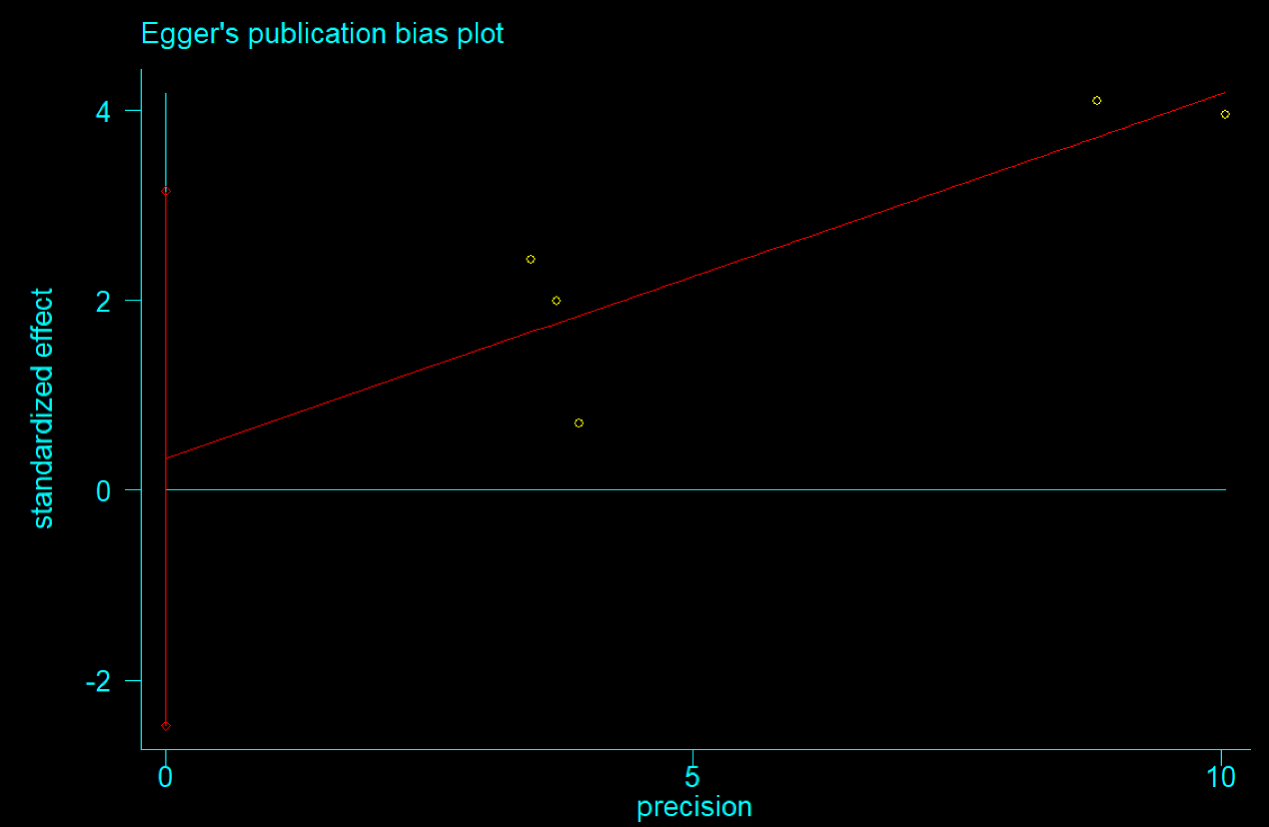


Supplementary Figure 6 Egger's funnel plot of TM6SF2 rs58542926 T/C in the allelic model


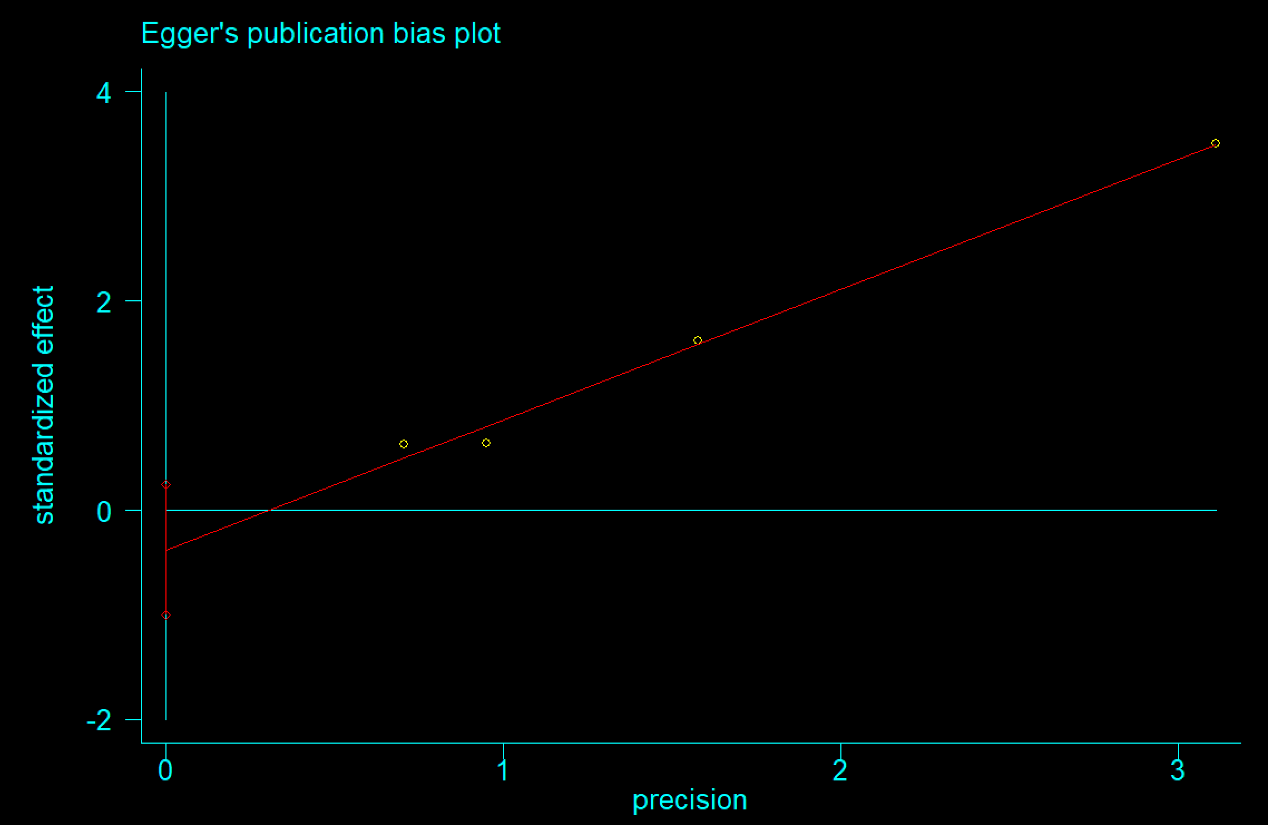


Supplementary Figure 7 Egger's funnel plot of TM6SF2 rs58542926 T/C in the recessive model


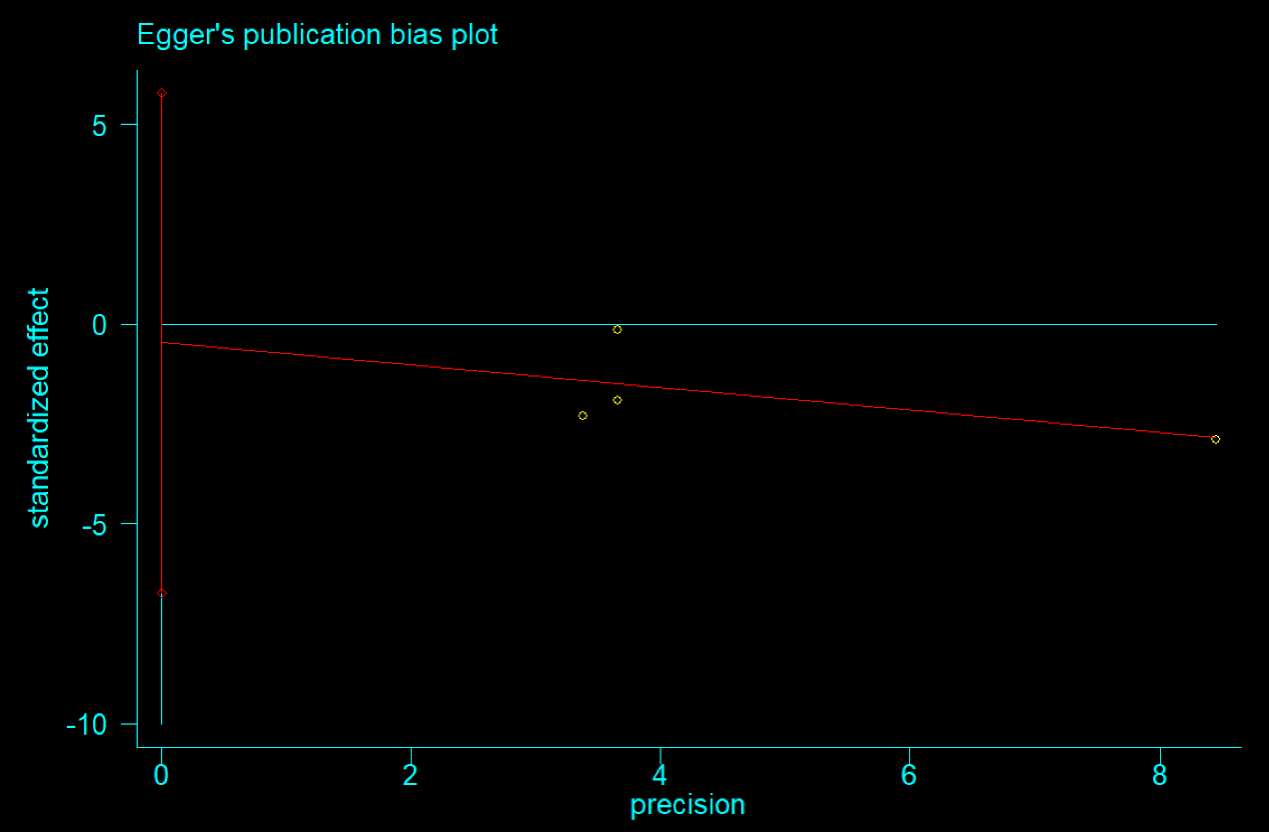


Supplementary Figure 8 Egger's funnel plot of TM6SF2 rs58542926 T/C in the super-dominant model
